# Computational simulation of aqueous humour dynamics in the presence of a posterior-chamber versus iris-fixed phakic intraocular lens

**DOI:** 10.1371/journal.pone.0202128

**Published:** 2018-08-13

**Authors:** José Ignacio Fernández-Vigo, Alfonso C. Marcos, Rafael Agujetas, José María Montanero, Inés Sánchez-Guillén, Julián García-Feijóo, Adrián Pandal-Blanco, José Ángel Fernández-Vigo, Ana Macarro-Merino

**Affiliations:** 1 Departamento de Oftalmología, Hospital Universitario Clínico San Carlos, Instituto de Investigación sanitaria San Carlos, Madrid (Spain); 2 Centro Internacional de Oftalmología Avanzada, Madrid (Spain); 3 Departamento de Expresión Gráfica, Universidad de Extremadura, Badajoz (Spain); 4 Departamento de Ingeniería Mecánica, Energética y de los Materiales and Instituto de Computación científica avanzada (ICCAEx), Universidad de Extremadura, Badajoz (Spain); 5 Escuela de Ingeniería, Universidad de Oviedo, Oviedo (Spain); 6 Facultad de Medicina, Universidad de Extremadura, Badajoz (Spain); Duke University, UNITED STATES

## Abstract

**Purpose:**

To compare aqueous humour (AH) dynamics in the presence of a precrystalline (Implantable Collamer Lens^®^; ICL) or iris-fixed (Artiflex^®^) phakic intraocular lens (PIOL).

**Methods:**

By computational fluid dynamics simulation, AH flow was modelled through a peripheral iridotomy (PI) or central lens hole (both 360 μm) in the presence of an Artiflex or ICL lens, respectively. The impacts of AH flow were then determined in terms of wall shear stress (WSS) produced on the endothelium or crystalline lens. Effects were also modelled for different scenarios of pupil diameter (PD 3.5 or 5.5 mm), ICL vault (100, 350, 800 μm) and number of Artiflex iridotomies (1 or 2) and location (12 or 6 o’clock).

**Results:**

For a PD of 3.5 mm, AH volumes flowing from the posterior to the anterior chamber were 37.6% of total flow through the lens hole (ICL) and 84.2% through PI (Artiflex). For an enlarged PD (5.5 mm), corresponding values were 10.3% and 81.9% respectively, so PI constitutes a very efficient way of evacuating AH. Central endothelial WSS in Pa was lower for the large vault ICL and the Artiflex (1^−03^ and 1.1^−03^ respectively) compared to the PIOL-free eye (1.6^−03^). Crystalline lens WSS was highest for the lowest vault ICL (1^−04^).

**Conclusions:**

AH flow varied according to the presence of a precrystalline or iris-fixed intraocular lens. Endothelial WSS was lower for an implanted ICL with large vault and Artiflex than in the PIOL-free eye, while highest crystalline WSS was recorded for the lowest vault ICL.

## Introduction

To correct high myopia, two of the most popular options are the placement of a posterior chamber (PC) or anterior chamber (AC) iris-fixed phakic intraocular lens (PIOL) such as the implantable collamer lens (ICL, STAAR Surgical AG, Nidau, Switzerland)^®^ [1,2] or Artiflex (Ophthec, The Netherlands)^®^, respectively [3,4].

Despite infrequent complications, these PIOLs have common potential risks such as ocular hypertension, pupillary block, pigment dispersion, endothelial damage, angle narrowing, and cataracts [3–10].

An implanted PIOL is an obstacle that hinders the passage of AH from the PC to the AC through the pupil, forcing the AH to redistribute. To prevent such AH flow complications, a peripheral iridotomy (PI) is performed before placement of an iris-fixed lens while the latest ICL model (V4c) has a central hole or aquaport for AH flow [11–13]. In effect, AH plays a key role in many complications. Thus, in the presence of a PIOL, reduced AH could directly cause ocular hypertension or mechanical trauma, and indirectly may lead to the development of cataract or endothelial damage by compromising oxygen and nutrient delivery to the cells.

To date the dynamics of AH flow[14–17] following the implant of a PIOL has been barely addressed.[18–21] Kawamorita et al. [18] suggest that AH flow on the anterior surface of the crystalline lens is improved by the presence of the central-hole in the ICL more than by an iridotomy. Repetto et al. [20] examined the response of the cornea and iris to AH flow effects produced after iris-fixed PIOL placement. Interestingly, Yamamoto et al. [22] showed that AH flow through a PI is affected by pupil diameter (PD) and this flow could have possible effects on the endothelium.

In the past few years, clinical studies have assessed the efficacy and safety of the more widely used PIOLs, posterior chamber and iris-fixed [23–26]. However, no previous study has compared AH flow dynamics in the presence these two PIOL types. The present study was designed to compare AH flow and the endothelial and crystalline stresses produced by this flow in the presence of these intraocular lenses in relation to factors such as PD, lens vault and number or location of iridotomies.

## Methods

The PIOLs numerically simulated were implantable collamer lens (ICL^®^, STAAR Surgical AG, Nidau, Switzerland) and Artiflex^®^ (Ophtec, The Netherlands).

The ICL is a foldable phakic lens made of collamer, a biocompatible, flexible, and absorbent material with a convex-concave central optic zone. This precrystalline lens is placed in the PC between the iris and crystalline lens and is supported by its haptics in the ciliary sulcus. The model employed was V4c which has a 360-micron hole in the middle of the optics, or KS-Aquaport (STAAR Surgical AG). This port facilitates AH flow from the PC to AC, avoiding the need for a PI as with previous ICL models. This PIOL was simulated using an 11 spherical diopter rigid lens.

Artiflex is a foldable AC iris-fixed lens with two haptics as claws. It is made of polysiloxane and has a convex-concave body. This lens was simulated for a single iridotomy of 360 μm at 12 or 6 o’clock, and a double iridotomy of 360 μm each at 10 and 2 o’clock.

**Fig 1:**
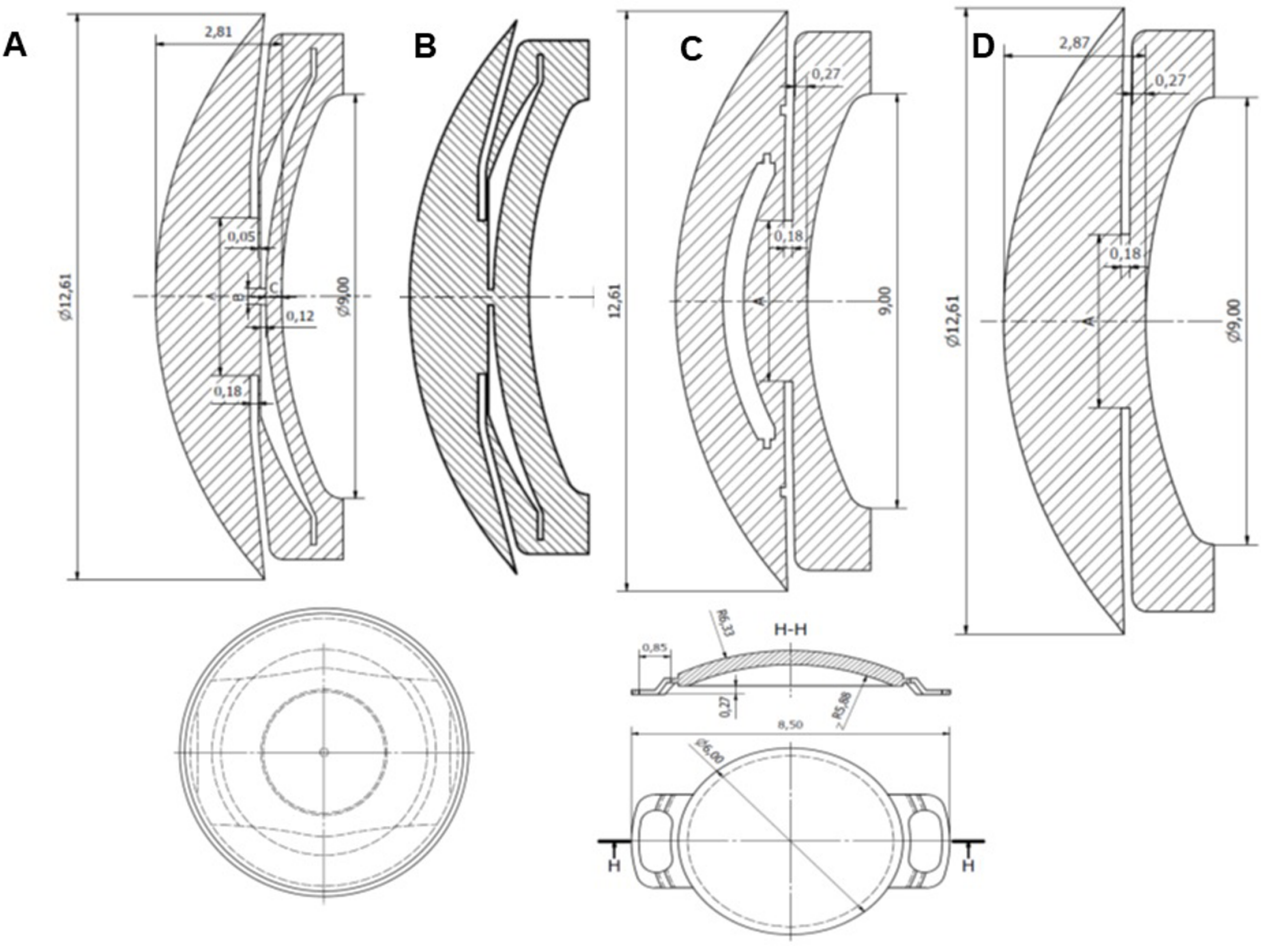
Standard measurements for a human eye used in the simulations. Anterior segment dimensions employed in the simulations and the phakic lens implanted: ICL (A), ICL vault 800 μm (B, note the anterior displacement of the lens), Artiflex (C) and normal eye without a phakic intraocular lens (D).

We also simulated an eye without a PIOL. Standard values for the adult human eye and the lenses used in the simulations are provided in Table 1. Fig 1 shows the dimensions of the anterior segment in eyes implanted with ICL or Artiflex and in the PIOL-free eye employed in the simulations.

**Table 1:** Variables included in the numerical simulation based on standard values for an adult human eye and implantable collamer lens (ICL) and Artiflex lens measurements.

Variable	Value
Anterior chamber diameter (mm)	12
Anterior chamber depth (mm)	3.20
Crystalline lens diameter (mm)	9.0
Crystalline lens thickness (mm)	4.0
Iris thickness (mm)	0.18
Distance between iris and crystalline (μm) for ICL	0.93
Distance between iris and crystalline (mm) for Artiflex and PIOL-free eye (in the center of the lens)	0.27
**ICL lens**	
Distance between iris and lens (mm)	0.12
Lens length (mm)	8.75
Lens width (mm)	3.50
Lens thickness (mm)	0.12
Central hole diameter (μm)	360
Vault = distance between crystalline and ICL lens (μm)	100; 350; 800
**Artiflex lens**	
Lens length (mm)	8.5
Lens width (mm)	6
Lens thickness (mm)	0.45
Iridotomy (microns)	360
**Other parameters**	Value
Linear expansion coefficient of aqueous humour b (K^-1^)	0.0003
Density of aqueous humour ρ_0_ (kg/m^3^)	998.2
Dynamic viscosity of aqueous humour μ (Pa·s)	0.001
Gravitational acceleration g (m/s^2^)	9.81
Thermal conductivity K (W/m·K)	0.6
Specific heat C_p_ (J/kg·K)	4182
Inflow surface area (mm^2^)	42.39
Outflow surface area (mm^2^)	86.75

mm = millimetres; kg = kilograms; m/s^2^ = metres per second.

### Numerical simulation

A three-dimensional full study simulation based on computational fluid dynamics (CFD) was performed with Ansys Fluent software (v16.2, ANSYS Inc. Pennsylvania, USA) enhancing our previous geometry model [19]. Simulations were conducted in steady laminar flow mode, setting a constant AH inflow rate of 2 μL/min (3.34 x 10–8 kg/s) and outflow boundary condition at the outlet section [19]. No-slip boundary conditions were prescribed on the solid surfaces. The behaviour of AH is Newtonian and incompressible. It was assumed that the fluid is removed through the trabecular meshwork at the same rate as it is produced. We did not consider the uveoscleral pathway, only the trabecular pathway as this accounts for 90% of AH removal. The temperature of the iris and the crystalline lens was set to 37°C. The temperature of the anterior cornea was set to 34°C when the eye is open, and 37°C when is closed (the eyelid covers the cornea). The temperature of the posterior corneal surface was calculated assuming a linear distribution across the cornea. The lenses were considered as adiabatic walls because their thermal conductivities are much smaller than that of AH. The buoyancy effects of the temperature gradient were modelled through the Boussinesq approximation assuming a fluid density change with temperature of: $\;\rho \;=\;\rho_{0}\left[1-\beta \left(T-T_{0}\right)\right]$. In a grid dependence analysis, we constructed three grids comprising coarse, medium or fine cells. Static pressure at a fixed point was used as the reference variable. When results for the fine and coarse grids were compared with those for the medium grid (7.5x10^5^), variation was under 1% so the medium grid was selected for the final calculations. WSS values lower than 10^−4^ Pa and 10^−7^ Pa could not be calculated reliably when the eye is open and closed, respectively.

Iridocorneal angle width was set at 30 degrees [10]. For this we had to modify the angle using the software to adjust it to the high vault. Although the eye is fairly symmetrical about its axis, this symmetry is broken by gravity, and consequently flow is three-dimensional. The effects of gravity were also taken into account in the simulations.

Dynamic viscosity of 0.001 Pa s described for a normal human eye was used, [14]. We examined the influence of the dynamic viscosity by considering the values 0.75^−3^ Pa s and 0.90^−3^ Pa s [27,28].

### Variables analysed

**Fig 2:**
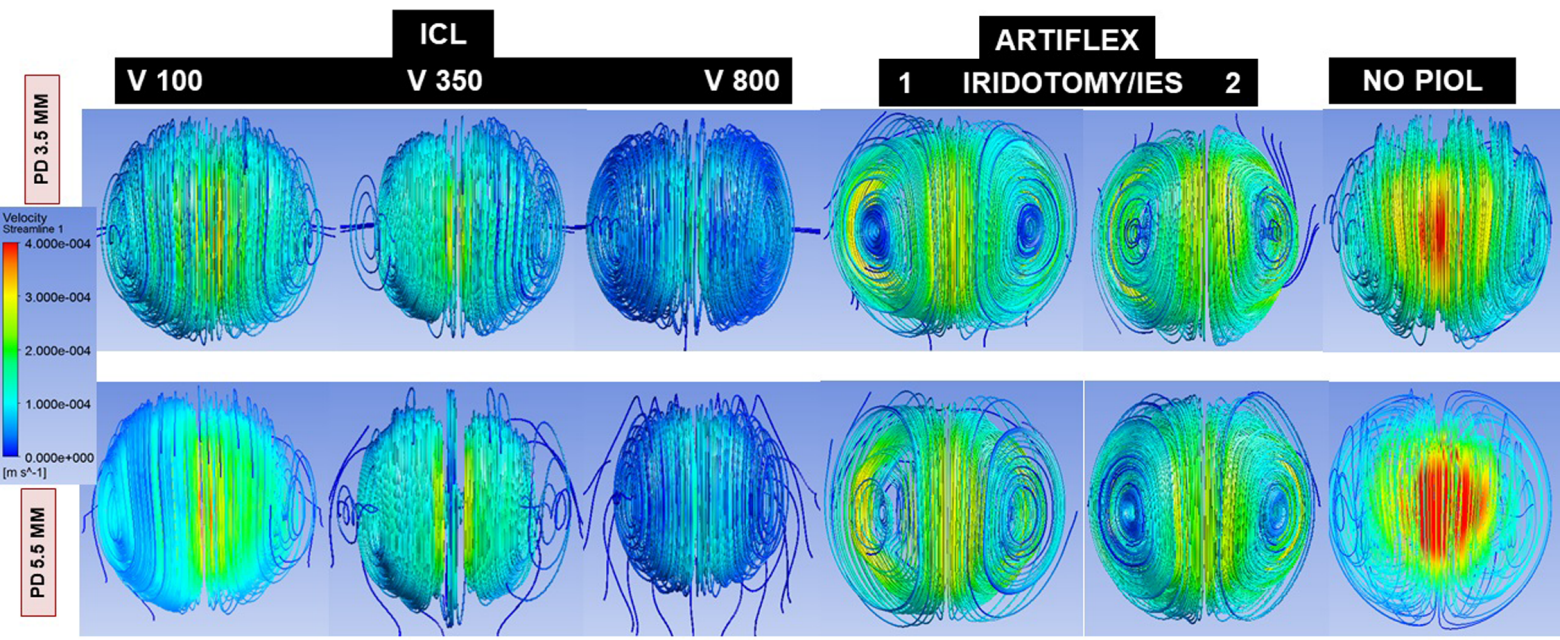
Streamlines of aqueous humor in the 12 scenarios modelled depending on the type of lens implanted (ICL, Artiflex or normal PIOL-free eye), pupil diameter (PD 3.5 to 5.5 mm), ICL vault (V 100, 350, 800) and number of iridotomies (1 or 2 for Artiflex). The colour represents the velocity magnitude.

The AH volume flowing through the passage ways tested (central hole in ICL and iridotomy in Artiflex) from the PC to AC were compared for 14 scenarios differing according to: lens type (ICL versus Artiflex), pupil diameter (PD, 3.5 or 5.5 mm), ICL vault (distance between the anterior surface of the crystalline lens and the posterior surface of the ICL) simulated as narrow (100 microns, V100), standard (350 microns, V350), or wide (800 microns, V800), and number (one or two) and location (12 or 6 o’clock) of iridotomies for Artiflex. Also, these situations were studied for both an open and a closed eye. To illustrate AH flow when the eye is open, the trajectory analysis presented in Fig 2 shows streamlines coloured by velocity magnitude, i.e., from when AH is synthesized in the ciliary body to when it exits via the trabecular meshwork. It may be seen a typical vortex due to the natural convection. S1 Fig shows the trajectory analysis in the closed eye.

**Fig 3:**
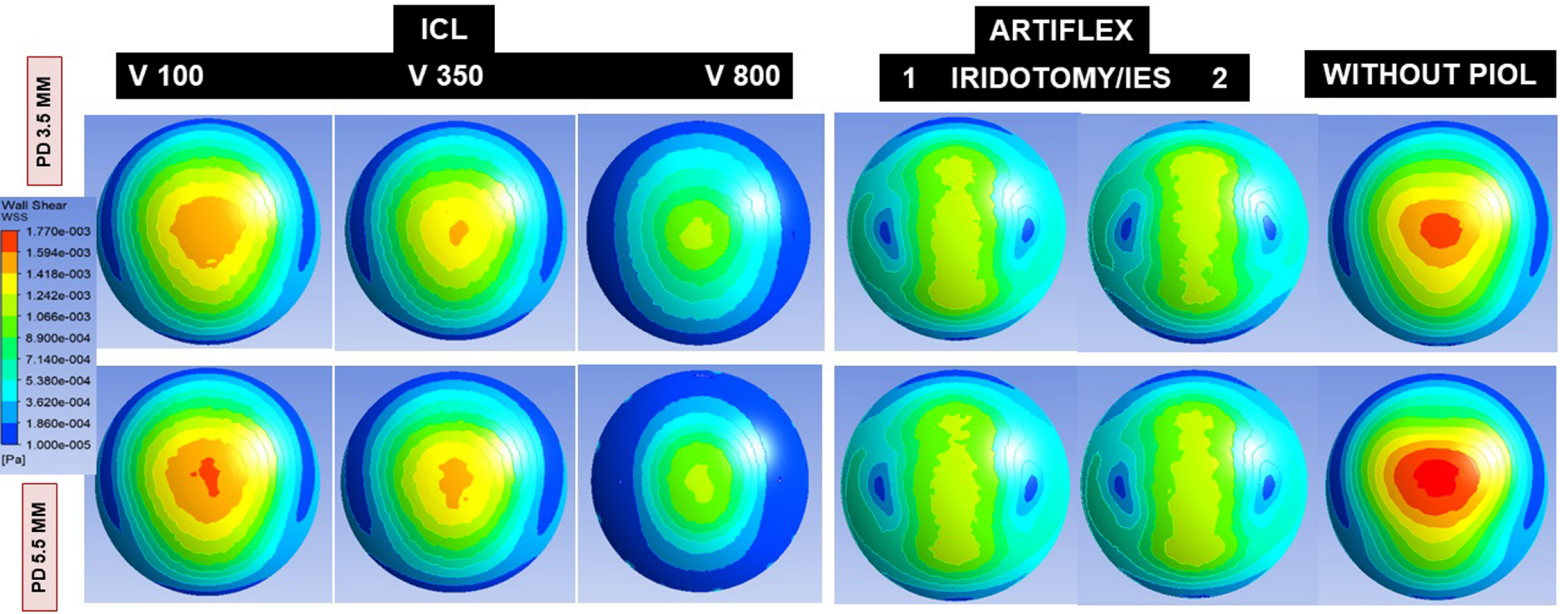
Wall shear stress on the corneal endothelium produced by aqueous humor flow through the central hole (ICL) or iridotomy (Artiflex) according to pupil diameter (PD = 3.5 to 5.5 mm), ICL vault (V = 100, 350, 800) and number of Artiflex iridotomies (1 or 2).

Wall shear stress (WSS) on the endothelium (Fig 3) was calculated for the whole corneal surface and for successive concentric rings: >11 mm, 9–11 mm, 7–9 mm, 5–7 mm, 3–5 mm, and central 3 mm.

**Fig 4:**
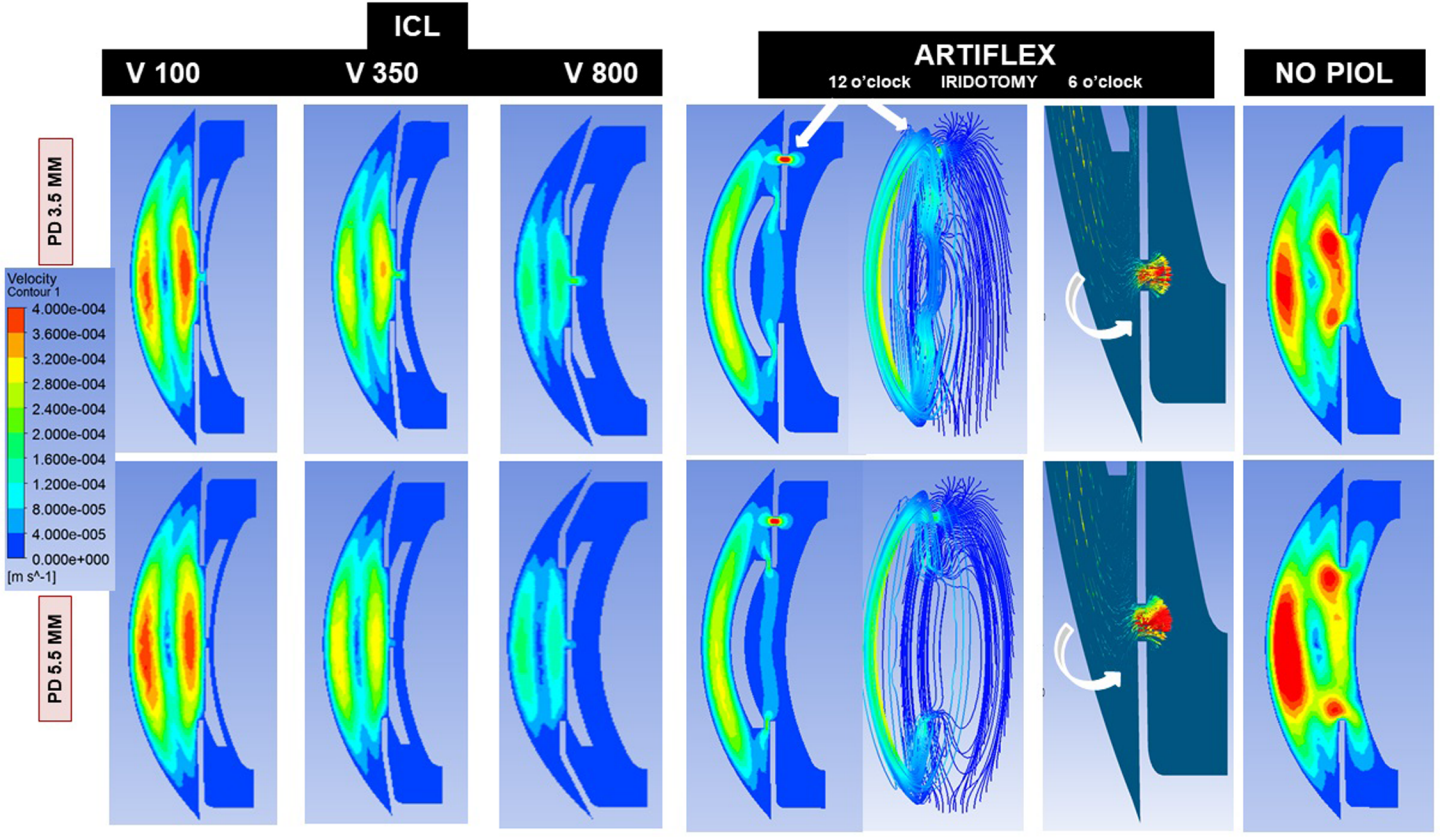
Velocity contours of AH flow through the central hole (ICL) or iridotomy (Artiflex) in the vertical plane according to pupil diameter (PD = 3.5 to 5.5 mm), ICL vault (V = 100, 350, 800) and number of Artiflex iridotomies (1 or 2).

**Fig 5:**
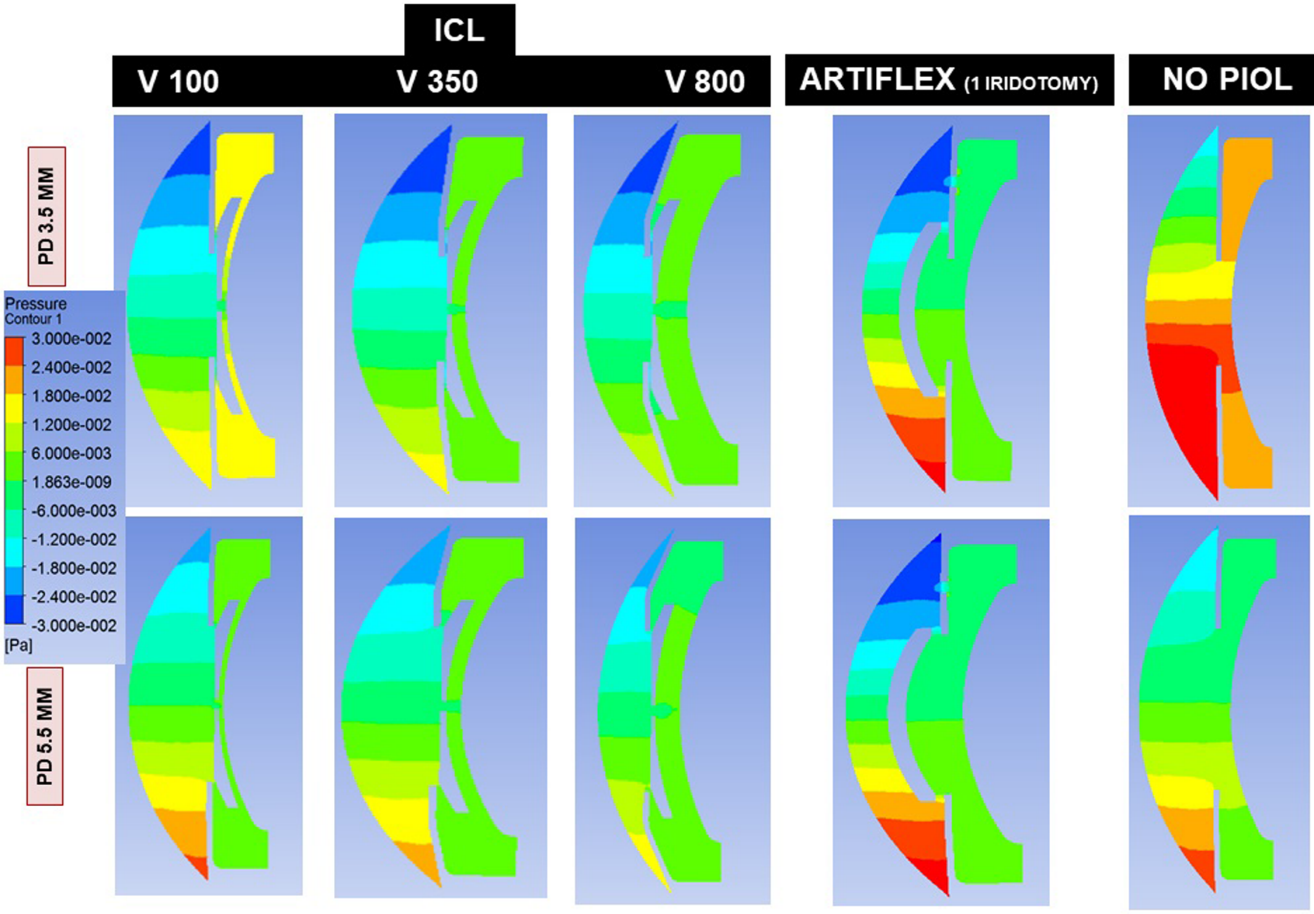
Pressure contours in the anterior segment through the central hole (ICL) or iridotomy (Artiflex) in the vertical plane according to pupil diameter (PD = 3.5 to 5.5 mm), ICL vault (V = 100, 350, 800) and number of Artiflex iridotomies (1 or 2).

Fig 4 shows AH velocity as it passes through the central ICL hole and through the PI (Artiflex), and Fig 5 shows the pressure distribution, along with its relationship with the anterior segment structures.

**Fig 6:**
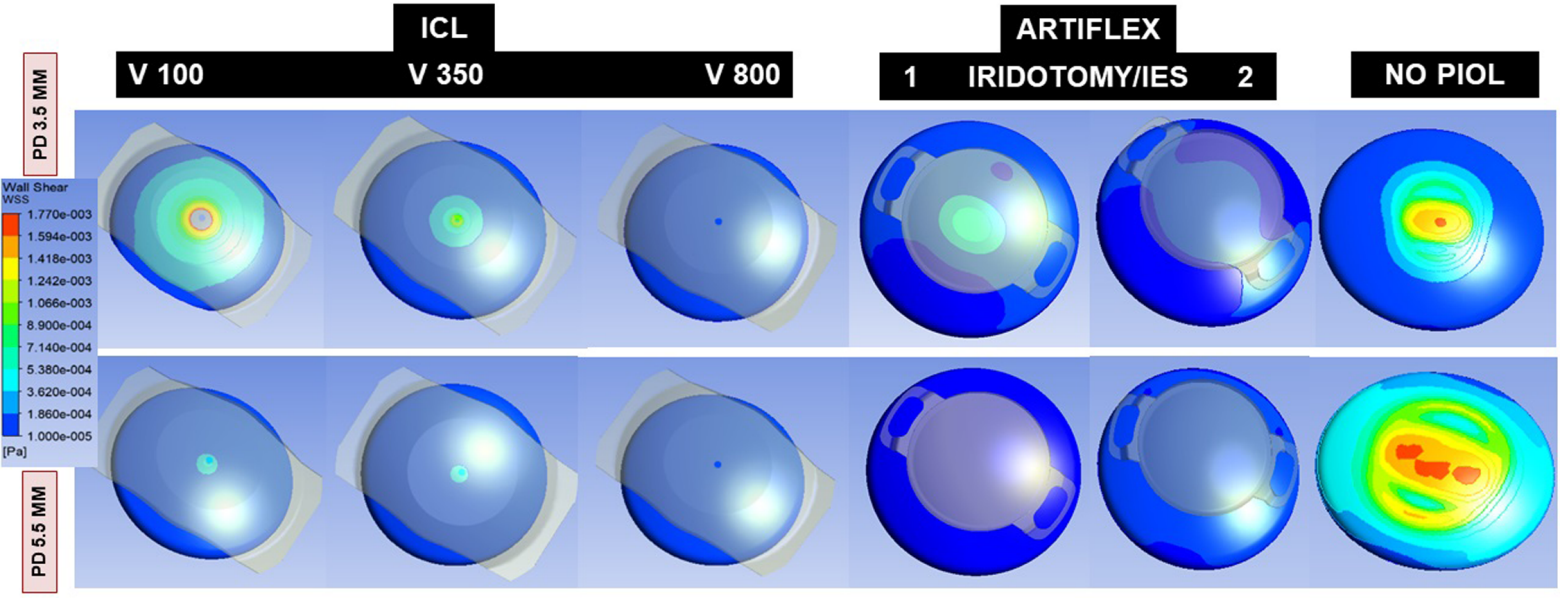
Wall shear stress on the crystalline lens produced by aqueous humor flow through the central hole (ICL) or iridotomy (Artiflex) according to pupil diameter (PD = 3.5 to 5.5 mm), ICL vault (V = 100, 350, 800) and number of Artiflex iridotomies (1 or 2).

WSS on the crystalline lens was also assessed (Fig 6). In the figure, the crystalline lens appears in blue and the PIOL is superimposed.

**Fig 7:**
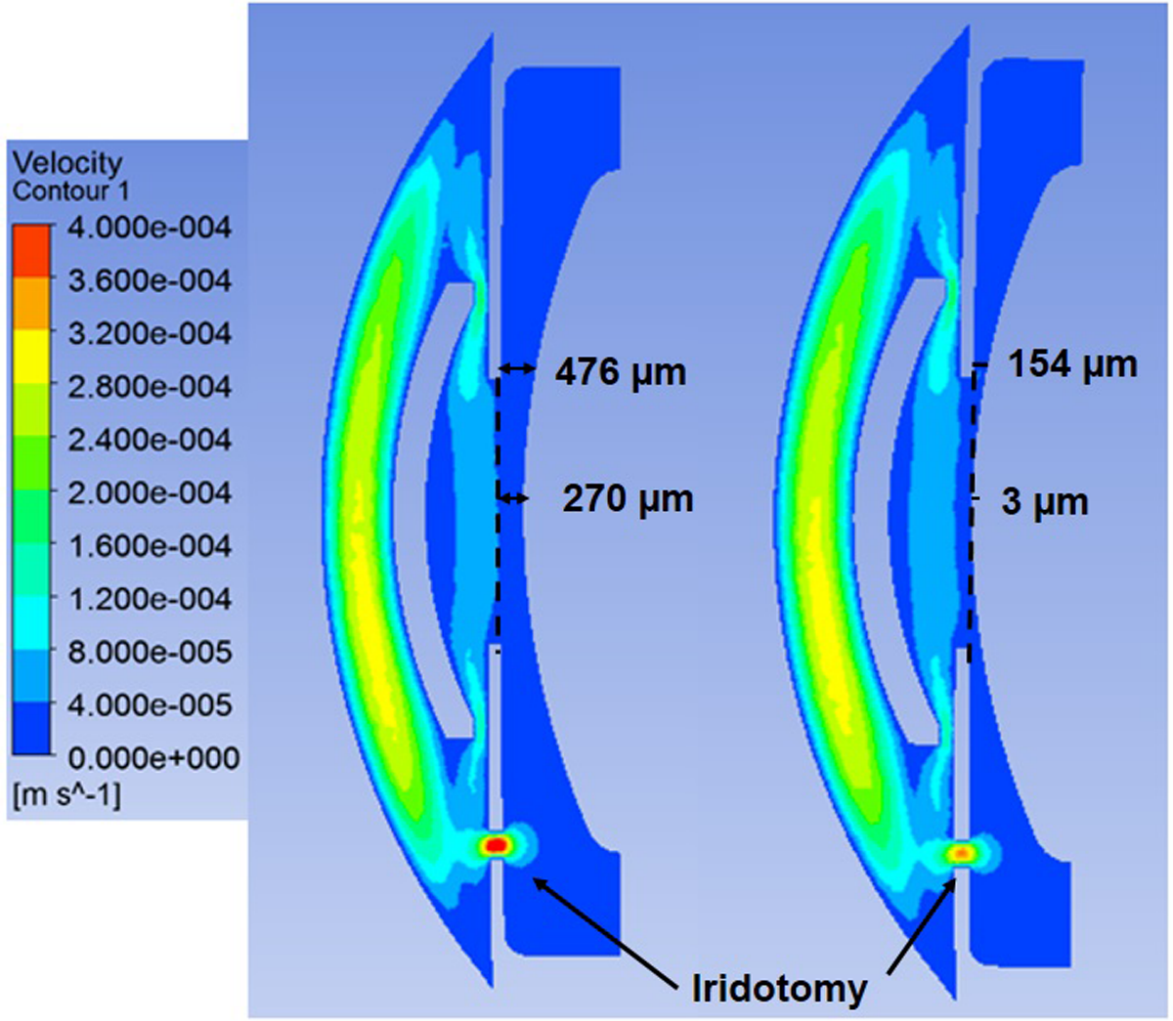
Velocity contours of aqueous humor flow in the vertical plane passing through an iridotomy at 6 o’clock from the anterior to the posterior chamber for the large (left) and small (right) crystalline-to-iris distance.

Also, the influence of the thickness of the passage between the iris and lens, in the Artiflex and the PIOL-free eye situations was calculated (270 μm versus 3 μm in the center of the lens; 476 μm versus 154 μm in the pupillary border; see Fig 7).

## Results

When the eye is open, the difference between the posterior cornea and iris temperatures is around 3°C and this creates buoyancy forces that drive the flow of AH in the AC. The liquid climbs over the (hot) iris and falls down next to the (cold) posterior cornea forming a vortex that occupies the whole chamber. The flow in the PC is almost isothermal and, therefore, much weaker, being essentially determined by the injection of AH through the ciliary body and the exit across the pupil. When the eye is closed, AH is essentially kept at the body temperature, and thermal convection does not occur, so the situation is completely different. In this section, we firstly describe the results obtained for an open eye, and then we mention the differences with respect to the closed eye case.

The simulations for an open eye show that a significant amount of liquid is evacuated through the central hole of the ICL lens from the PC to the AC (ICL, V350 and PD 3.5 mm), being 37.6% of the total flow rate (i.e., 62.4% passed through the pupil). This percentage was notably reduced to 10.3% when the pupil was enlarged to 5.5 mm. That flow rate plunges when the pupil dilates because the hydraulic resistance of the channel formed by posterior iris surface and anterior ICL surface sharply decreases in that case, which means that most of AH volume can pass directly through the pupil. For the Artiflex lens, 84.2% passed through the PI and this proportion fell slightly to 81.9% when the pupil was enlarged. In the double iridotomy scenario, AH volume was 95.5% through both iridotomies and diminished to 89.3% for a PD of 5.5 mm. So, PI constitutes a very efficient way of discharging AH from the PC to the AC. This occurs due to the pressure reduction taking place in front of the PI due to thermal convection in the AC (Fig 5). In fact, if the iridotomy is at 6 o’clock, the opposite effect is produced: the flow is inverted, passing 79% of AH flow from the AC to the PC, due to the increase of pressure in front of the PI associated to thermal convection in the AC (Fig 5).

Our data indicate that the reduction in AH flowing through the channels produced when the pupil enlarges was proportionally larger for the ICL than Artiflex lens, also in the ICL V800 scenario (39.6% of the AH volume drop to 14.1%), being more drastic (16 times; 27.6% to 1.7%) in the low vault ICL. The explanation for this could be that for a narrow vault the resistance will increase due to the considerably narrowed space.

WSS on the whole endothelial surface (Table 2 and Fig 3) was lower for the scenario ICL V800 (3^−04^ Pa) compared with the remaining situations: ICL V100, the PIOL-free eye and the Artiflex (7^−04^ Pa for all). Central endothelial WSS was lower for ICL V800 and for Artiflex (1.0^−03^ Pa and 1.1^−03^ Pa respectively) than for ICL V100 or PIOL-free eye (1.5^−03^ Pa and 1.6^−03^ Pa). Peripheral endothelial WSS (ring 7–9 mm) was highest also for the ICL V100 (1^−03^ Pa) being similar compared with the PIOL-free eye (1^−03^ Pa), than for the V800 scenario (5^−04^ Pa), and 1.25 times greater compared with Artiflex (1 iridotomy, PD 3.5 mm) (8^−04^ Pa).

**Table 2:** Aqueous humor volume and Wall shear stress (WSS) produced on the endothelium and crystalline lens for the 14 scenarios modelled according to pupil diameter and the phakic intraocular lens (PIOL) implanted (Implantable collamer lens, ICL; Artiflex; or normal PIOL-free eye) in an open eye (taking into account the convection).

Open eye	ICL vault (μm)						Artiflex						PIOL-free eye	
Parameter	100		350		800		1 iridotomy (12 o’clock)		1 iridotomy (6 o’clock)		2 iridotomies			
Pupil diameter (mm)	3.5	5.5	3.5	5.5	3.5	5.5	3.5	5.5	3.5	5.5	3.5	5.5	3.5	5.5
Flow through central hole or iridotomy (%)	27.6	1.70	37.6	10.3	39.6	14.1	84.2	81.9	82	79	95.5	89.3	N/A^a^	N/A^a^
Volume through central hole or iridotomy (m^3^/s)	9.23^−12^	5.68^−13^	1.26^−11^	3.44^−12^	1.32^−11^	4.70^−12^	2.82^−11^	2.74^−11^	2.75^−11^	2.65^−11^	3.18^−11^	2.99^−11^	N/A^a^	N/A^a^
WSS whole cornea (Pa)	7^−4^	7^−4^	6^−4^	6^−4^	4^−4^	3^−4^	7^−4^	7^−4^	7^−4^	7^−4^	7^−4^	7^−4^	7^−4^	8^−4^
WSS ring >11 mm (Pa)	3^−4^	3^−4^	3^−4^	2^−4^	2^−4^	1^−4^	5^−4^	5^−4^	5^−4^	5^−4^	5^−4^	5^−4^	3^−4^	3^−4^
WSS ring 9–11 mm (Pa)	8^−4^	8^−4^	6^−4^	6^−4^	4^−4^	2^−4^	7^−4^	6^−4^	7^−4^	6^−4^	7^−4^	7^−4^	8^−4^	8^−4^
WSS ring 7–9 mm (Pa)	1.0^−3^	1.0^−3^	9^−4^	8^−4^	5^−4^	4^−4^	8^−4^	7^−4^	7^−4^	7^−4^	7^−4^	8^−4^	1.0^−3^	1.1^−3^
WSS ring 5–7 mm (Pa)	1.2^−3^	1.3^−3^	1.1^−3^	1.1^−3^	7^−4^	7^−4^	9^−4^	9^−4^	9^−4^	9^−4^	9^−4^	9^−4^	1.2^−3^	1.4^−3^
WSS ring 3–5 mm (Pa)	1.4^−3^	1.5^−3^	1.2^−3^	1.3^−3^	9^−4^	9^−4^	1.0^−3^	1.0^−3^	1^−3^	1^−3^	1.0^−3^	1.0^−3^	1.4^−3^	1.6^−3^
WSS ring <3 mm (Pa)	1.5^−3^	1.6^−3^	1.4^−3^	1.4^−3^	1.0^−3^	1.0^−3^	1.1^−3^	1.1^−3^	1.1–3	1.1–3	1.1^−3^	1.1^−3^	1.6^−3^	1.8^−3^
WSS crystalline lens (Pa)	1^−4^	<10^−4^	<10^−4^	<10^−4^	<10^−4^	<10^−4^	<10^−4^	<10^−4^	<10^−4^	<10^−4^	<10^−4^	<10^−4^	<10^−4^	7^−4^

^a^N/A: Not applicable

So, the highest corneal WSS quantified was for the PIOL-free eye or the physiological condition. The large difference between the flow intensities in the AC and PC also explains why the central hole of the ICL lens, or the peripheral iridotomies made when Artiflex lens is implanted, does not considerably influence the magnitude and spatial distribution of the WSS in the cornea. The presence of an Artiflex lens in the AC does substantially modify the flow pattern in that region. The buoyancy-driven vortex breaks into two smaller ones, located symmetrically with respect to the AC mid-plane. This effect changes the distribution of WSS over the posterior corneal surface.

Fig 4 shows the flow velocity in the different scenarios. For ICL V350 and V800 the increased flow velocity of the jet through the central hole was dissipated in the AC, neutralized by the convection, and did not affect the endothelium. However, in the V100, this AH stream could impact on the central endothelium, as quantified by WSS. This increase of WSS in this case can also be explained in terms of the variation of the temperature field due to ICL. Lowest central endothelial WSS was recorded for the ICL V800 (1^−03^ Pa), because the WSS decreases monotonously as the vault increases. For instance, the WSS at the central endothelium decreases about 50% of its value when the vault increases from 100 to 800 μm. This effect is probably caused by changes in the iris geometry due to the increment of the vault, rather than by the ICL lens itself.

The stream of AH through a PI could increase the magnitude of WSS in the peripheral endothelium. This does not occur because that stream is neutralized by the convection.

Regarding the peripheral (9–11 mm) and central (<3 mm) endothelial WSS, they were lower for ICL V800. It should be noted that PD affected more the distribution of endothelial WSS for ICL than for Artiflex, for which WSS representations and values were similar irrespective of PD (Fig 3 and Table 2).

WSS on the crystalline lens was highest for ICL V100 (1^−04^ Pa) and a small PD, than in the other situations (Fig 6), followed by the case of ICL V350 and same PD. In the remaining situations, it may be observed that no WSS increase was produced. In this figure, it may also be seen that PD is an important determinant for ICL V100 since as the pupil enlarges, WSS on the crystalline lens drops to nearly normal values.

When the eye is closed, AH is essentially kept at the body temperature, and thermal convection does not occur, so the situation is completely different (see Table 3). In this case, the liquid in the AC flows in the radial direction from the pupil towards the trabecular pathway at a very small speed. In the presence of an Artiflex lens, the volume that flows through the PI is the same irrespective of its location, superior or inferior. The flow in the PI diminishes from 84.2% when the eye is open to 6.1% when the eye is closed, and it increases through the central hole of the ICL from 37.6% to 82.2%. In addition, the resulting WSS on both the endothelium and the lens becomes negligible when the eye is closed.

**Table 3:** Aqueous humor volume and Wall shear stress (WSS) on the endothelium and crystalline lens for the 14 scenarios modelled according to pupil diameter and the phakic intraocular lens (PIOL) implanted (Implantable collamer lens, ICL; Artiflex; or normal PIOL-free eye) in a closed eye (without convection).

Closed eye	ICL vault (μm)						Artiflex						PIOL-free eye	
Parameter	100		350		800		1 iridotomy (12 o’clock)		1 iridotomy (6 o’clock)		2 iridotomies			
Pupil diameter (mm)	3.5	5.5	3.5	5.5	3.5	5.5	3.5	5.5	3.5	5.5	3.5	5.5	3.5	5.5
Flow through central hole or iridotomy (%)	73.4	8.6	88.2	20.2	89.7	22.6	6.1	3.2	6.1	3.2	11.6	6.6	N/A^a^	N/A^a^
Volume through central hole or iridotomy (m^3^/s)	2.46^−11^	2.88^−12^	2.95^−11^	6.76^−12^	3.00^−11^	7.6^−12^	2.05^−12^	1.06^−12^	2.05^−12^	1.06^−12^	3.89−^12^	2.20^−12^	N/A^a^	N/A^a^
WSS whole cornea (Pa)	1.30^−05^	1.30^−05^	1.68^−05^	2.01^−05^	3.35^−05^	7.1^−05^	1.23^−05^	1.23^−05^	1.23^−05^	1.23^−05^	1.22^−05^	1.22^−05^	1.28^−05^	1.30^−05^
WSS ring >11 mm (Pa)	2.37^−05^	2.46^−05^	3.08^−05^	3.82^−05^	6.05^−05^	1.3^−04^	2.47^−05^	3.65^−07^	2.47^−05^	3.65^−07^	2.47^−05^	2.46^−05^	2.36^−05^	2.46^−05^
WSS ring 9–11 mm (Pa)	3.77^−06^	4.02^−06^	5.00^−06^	6.1^−06^	1.07^−05^	2.47^−05^	2.88^−06^	5.54^−07^	2.88^−06^	5.54^−07^	2.77^−06^	3.16^−07^	3.75^−06^	3.95^−06^
WSS ring 7–9 mm (Pa)	3.14^−06^	3.37^−06^	4.15^−06^	4.84^−06^	9.41^−06^	1.44^−05^	8.63^−07^	3.00^−06^	8.63^−07^	3.00^−06^	7.73^−07^	3.28^−07^	3.15^−06^	4.43^−07^
WSS ring 5–7 mm (Pa)	3.20^−06^	2.31^−06^	4.09^−06^	3.32^−06^	8.70^−06^	8.13^−06^	5.60^−07^	2.47^−05^	5.60^−07^	2.47^−05^	4.72^−07^	4.06^−07^	3.23^−06^	1.30^−06^
WSS ring 3–5 mm (Pa)	3.52^−06^	1.00^−06^	4.68^−06^	1.58^−06^	8.95^−06^	2.54^−06^	4.46^−07^	8.50^−07^	4.46^−07^	8.50^−07^	4.31^−07^	7.43^−07^	3.17^−06^	2.47^−06^
WSS ring <3 mm (Pa)	3.00^−06^	3.91^−07^	4.61^−06^	1.06^−06^	1.07^−05^	2.57^−06^	3.93^−07^	4.17^−07^	3.93^−07^	4.17^−07^	4.24^−07^	2.89^−06^	2.05^−06^	3.24^−06^
WSS crystalline lens (Pa)	2.6^−04^	3.1^−05^	6.9^−05^	1.6^−05^	2.0^−06^	4.4^−06^	1.7^−05^	8.3^−06^	1.7^−05^	8.3^−06^	1.6^−05^	8.1^−06^	9.0^−06^	9.0^−06^

^a^N/A: Not applicable

It was observed a significant influence of the viscosity value on the flow pattern. In the present study, as described by Fitt. et al. for a normal human eye, a dynamic viscosity of μ = 0.001 Pa s was used [14]. The percentage of flow that crosses the PI varies from 84% for the μ = 1^e-3^ Pa s [14], to 108% (a small reverse flow takes place in the pupil) for the μ = 0.75^e-3^ Pa s [27], 93% for the mu = 0.90^e-3^ Pa s [28].

Regarding the influence of the thickness of the passage between the iris and lens, reducing that distance from 476 μm to 154 μm considerably increases the pressure in the PC, but hardly affects the characteristics of the buoyancy flow in the AC. In fact, our simulation shows an increase by a factor around 7.5 of the pressure drop between the PC inlet section and the center of the pupil, while the WSS on the cornea remained practically the same.

The thickness of the passage between the iris and crystalline can significantly affect the flow crossing the iridotomy, especially the reverse flow taking place through the iridotomy located at 6 o’clock. If the iridotomy is located at 12 o’clock, the flow rate crossing that orifice increases from 84.2% to 85.2%. This increase is very small because most part of the AH volume already flows through the iridotomy even with the thick passage is considered. When the iridotomy is located at 6 o’clock, the increase of pressure in the PC reduces the reverse flow from 79% to 60.5%. Fig 7 shows the velocity magnitude in the vertical plane calculated in the presence of an iridotomy at 6 o’clock with the large (left) and small (right) crystalline-to-iris distance.

## Discussion

Both posterior chamber and iris-fixed PIOLs seem satisfactory options to treat high myopia [1–4,10,23–25], with no differences in efficacy or safety detected in a recent meta-analysis.[26] Our findings point to different AH flow behaviour and different WSS produced by this flow on the endothelium and crystalline lens depending on the type of lens implanted.

The AH volume flowing through the ICL central hole varied from 1.7 to 39.6% of the total volume (depending on the vault and PD), and from 79 to 95.5% through the PI of the iris-fixed Artiflex lens (according to the presence of one or two iridotomies), so PI constitutes the most efficient way of discharging AH from the PC to the AC. Pupil size was also found to largely condition AH behaviour, in the ICL more than in the Artiflex. This meant that for a pupil enlarged from 3.5 to 5.5 mm, the fluid volume flowing through the central port in the ICL diminished to ≤14.1%, and for the PI in Artiflex dropped to 81.9%. Thus, in the case of pupillary block (because of full contact between PIOL lens and iris), the PI could be more effective in preventing IOP elevations, showing that a single iridotomy seems effectively enough, so a double iridotomy is not necessary in terms of fluid dynamics.

In agreement with our results (Fig 4), Dvoriashyna et al.[29] described that most of AH flows through the PI, with almost all of it going through the PI for diameters larger than 150 μm. They described that the ideal size and location of a PI are influenced by various geometrical and fluid mechanical factors, the most relevant of which are the size of the hole, also analyzed by our group in a previous paper [19], and the length and height of the iris–lens channel. On the contrary, the location of the iridotomy only changes the total flow by 2% from an iridotomy placed in the middle of the PC to one placed peripherally, which is because the pressure is approximately uniform across the PC.

In our study, if the PI is located at 6 o’clock, the flow is inverse, passing 79% from the AC to the PC. This is one of the reasons why many ophthalmologists perform an inferior PI in the pigmentary dispersion syndrome, to reduce the gradient differences between AC and PC, and to solve the iris concavity. Accordingly, Dvoriashyna et al. noted that, possibly in response to pupillary movements, the direction of flow through the PI would reverse and the jet of AH will impact on the crystalline lens, and could cause cataract related to the possible occurrence of large values of the WSS on the lens opposite to the iridotomy [29]. Based on our results, we consider that it is highly unlikely that this magnitude of WSS could damage the lens.

Previous papers have reported thicknesses of the passage between the iris and crystalline much smaller than the one considered in this work. For instance, Silver and Quigley considered crystalline lens-to-iris minimum distances between 3 and 7 μm [30]. In our study in the physiological case, the reduction of the passage between the iris and crystalline (from 476 μm to 154 μm) considerably increases the pressure in the PC, but hardly affects the characteristics of the buoyancy flow in the AC, while the WSS on the cornea remained practically the same.

In the Artiflex the flow crossing the iridotomy could be significantly affected by the thickness of that passage, especially the reverse flow taking place through the iridotomy located at 6 o’clock, due to the increase of pressure in the PC that reduces the reverse flow from 79% to 60.5%.

Besides the risks of the pupillary block and ocular hypertension [31] two other main clinical concerns regarding the use of a PIOL are endothelial damage and cataract [24–26,32–34].

Endothelial cell loss related to the implant of an ICL has been estimated at 6.5% surgically induced during the first year, and an average yearly loss rate of 1.2% after that [23]. In a recent clinical study, Jonker et al.[25] described in myopic patients implanted with the iris-fixed PIOL significant linear chronic endothelial cell loss of 16.6% and 21.5% from 6 months to 10 years postoperatively. This indicates that the endothelial cell threshold should be carefully determined for subsequent safe combined PIOL removal and cataract surgery.

It is generally thought that in terms of inducing damage to the endothelium, an AC lens could be more dangerous than a PC lens. In our simulations, no significant differences were observed between this PC and AC lenses. Interestingly, the WSS produced on the whole and central endothelium was lower for an ICL V800 and Artiflex than in the normal PIOL-free eye. It has been hypothesized that the loss of endothelial cells could be due to an increase of the WSS on the cornea, however, Repetto et al. aimed that this speculation is not supported by the results of their study, thus alternative mechanisms should be invoked to explain this clinical finding [20]. In a pig model, Kaji et al.[35] determined a WSS greater than 0.1 Pa as the critical point at which cells start to detach from the cornea. In our simulations, WSS in the presence of both lenses was much lower than this value yet still within physiologically acceptable limits, as also observed by Konghar et al.[21] in a simulation for a perforated Artiflex. We think that, probably, only in the case of a large inflammation in uveitis or after an eye trauma with synechias around the pupil and in the PI or the central hole of the lens, the WSS could reach higher stresses values on the cornea. However, the level of WSS at which corneal endothelial damage would be sustained is unknown.[29]

These lenses are a barrier for AH flow, as them shields the pupil. We hypothesized that this reduction in the WSS in the presence of the PIOLs comparing with the PIOL-free eye or the physiological condition could probably indicate a compromised supply of nutrients delivery to the endothelium, rather than cell damage due to mechanical trauma. In agreement with our findings, Repetto et al.[20] observed that WSS on the cornea was lower in the presence than absence of an iris-fixed lens and suggested that the PIOL shielded the central cornea and could induce a lower rate of oxygen and nutrient supply.

In our simulation models, pupil size did not emerge as a major determinant of endothelial WSS, affecting, and only slightly, in the case of the ICL. Yamamoto et al.[22,36] demonstrated that pupil constriction leads to a marked increase in AH flow through the PI against the corneal endothelium, producing mechanical stress that is likely responsible for the peripheral corneal decompensation that sometimes occurs after a PI, especially in eyes with shallow AC. Dvoriashyna et al.[29] described that for certain PI diameters, the jet velocity through the PI might be large enough to cause possible corneal damage. In the present work, we also observed that increasing the flow rate through the PI may affect the peripheral endothelium, especially in the case of smaller pupils, but those WSS values are unlikely to damage the endothelium.

In the open eye there is a double vortex of streamlines due to the convection, very different from the radial streamlines that would be observed in the closed eye. Moreover, the main difference is that in the open eye much larger flow that starts on the opposite side to the iridotomy hole ends up draining into the PI. In a report by Kawamorita et al.[18] they suggested that the central-hole ICL leads to improved AH circulation in the front of the crystalline lens, being more physiological or homogeneous the circulation. However, they did not account for the effect of thermal gradients, which is the main mechanism for AH flow in an open eye. On the contrary, we have observed that the jet through the central hole is dissipated by the convection in the AC.

The other main concern related to the use of a PIOL is the development of cataract because candidates for these lenses are usually young patients with clear crystalline lenses. Several studies have shown that a narrow vault ICL increases the risk of cataract formation [24–26]. In the present study, we also observed that crystalline WSS was highest for ICL V100, but in larger pupil size, dropped to nearly normal values. However, in the remaining scenarios, WSS was not increased compared to the physiological condition of the PIOL free eye. Thus, different to the vault, PD seems not to be a determinant of crystalline WSS, such that in the presence of a very narrow or wide vault, it is better to have a large PD to minimize the stress produced on the crystalline lens.

The main limitation of our study was that it was based on a simulated theoretical model as it is difficult to measure AH dynamics in the human eye *in vivo*. Thus, we should interpret our findings with caution as theoretical results will always have inherent limitations [14–21]. The results are likely to depend significantly on the geometrical characteristics of the models. As an ophthalmologist, we cannot modify some variables such as the convection, the pressure contour or the viscosity of the AH, but we could decide the type of lens implanted and the location and size of the iridotomy, and we could also choose between different sizes of the lens that could change the dimensions of the anterior segment (the iris-lens channel depth, vault, anterior chamber angle) and consequently the AH distribution. Despite expected bias, however, bioengineering simulations can improve our understanding of ocular physiology and changes produced in response to surgery.

This is the first study to simulate AH dynamics in response to the presence of two types of phakic lens, a posterior chamber ICL lens versus an Artiflex iris- fixed lens. Several factors affecting AH dynamics were explored such as different-sized vaults following ICL implant, the presence of one or two and the location of the iridotomies in the case of the Artiflex lens and different pupil size. More work is needed to confirm our findings and further our understanding of the changes produced following the implant of different PIOLs including impacts produced on AH dynamics, especially in terms of alterations to the metabolism of anterior segment structures.

In conclusion, AH flow varies depending on the type of phakic lens implanted, ICL or Artiflex. AH flow varied according to the presence of a precrystalline or iris-fixed intraocular lens. PI constitutes a very efficient way of evacuating AH. Endothelial WSS was lower for an implanted ICL with large vault and Artiflex than in the PIOL-free eye, while highest crystalline WSS was recorded for the lowest vault ICL.

## Supporting information

S1 Fig Streamlines of AH in the 12 scenarios modelled depending on the type of lens implanted (ICL, Artiflex or normal PIOL-free eye), pupil diameter (PD 3.5 to 5.5 mm), ICL vault (V 100, 350, 800) and number of iridotomies (1 or 2 for Artiflex). The colour represents the velocity magnitude.
(PDF)
