# Supplementary material for: Computational simulation of aqueous humour dynamics in the presence of a posterior-chamber versus iris-fixed phakic intraocular lens
Source: PLoS One. 2018 Aug 13;13(8):e0202128. doi: 10.1371/journal.pone.0202128 (PMC6089426; doi:10.1371/journal.pone.0202128)
Supplement: S1 Fig — The colour represents the velocity magnitude. (PDF) [file pone.0202128.s001.pdf]

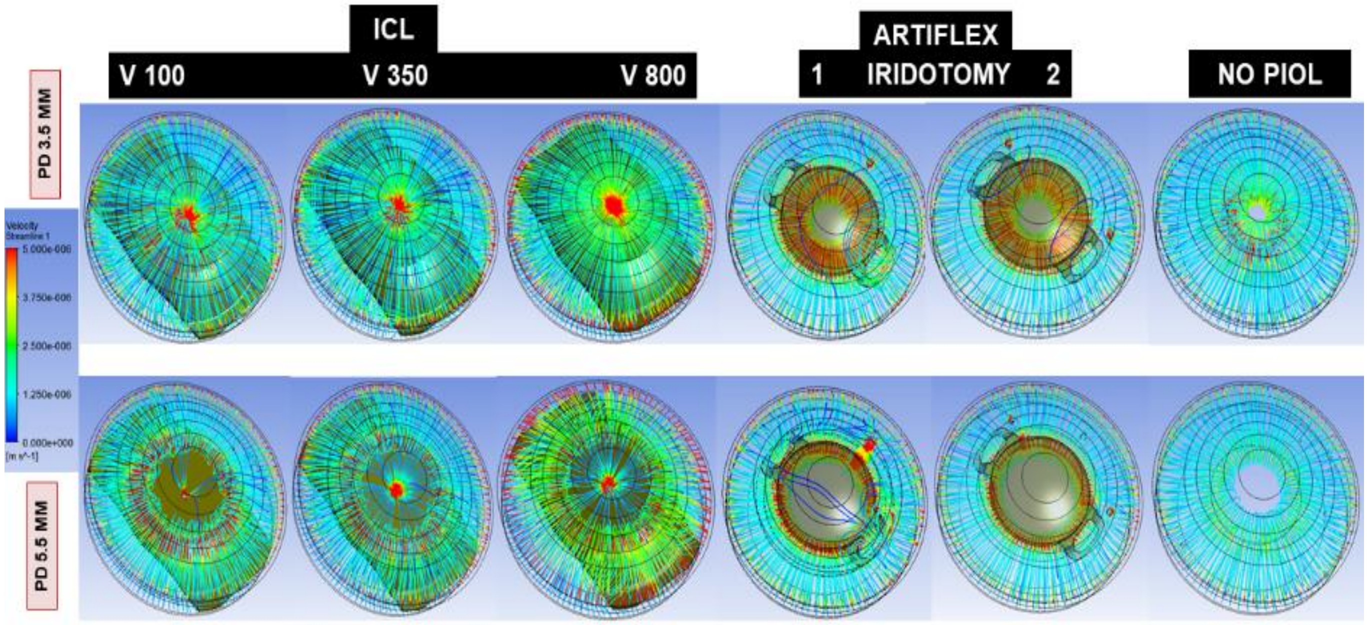

S1 Fig: Streamlines of AH in the 12 scenarios modelled depending on the type of lens implanted (ICL, Artiflex or normal PIOL-free eye), pupil diameter (PD 3.5 to 5.5 mm), ICL vault (V 100, 350, 800) and number of iridotomies (1 or 2 for Artiflex). The colour represents the velocity magnitude.

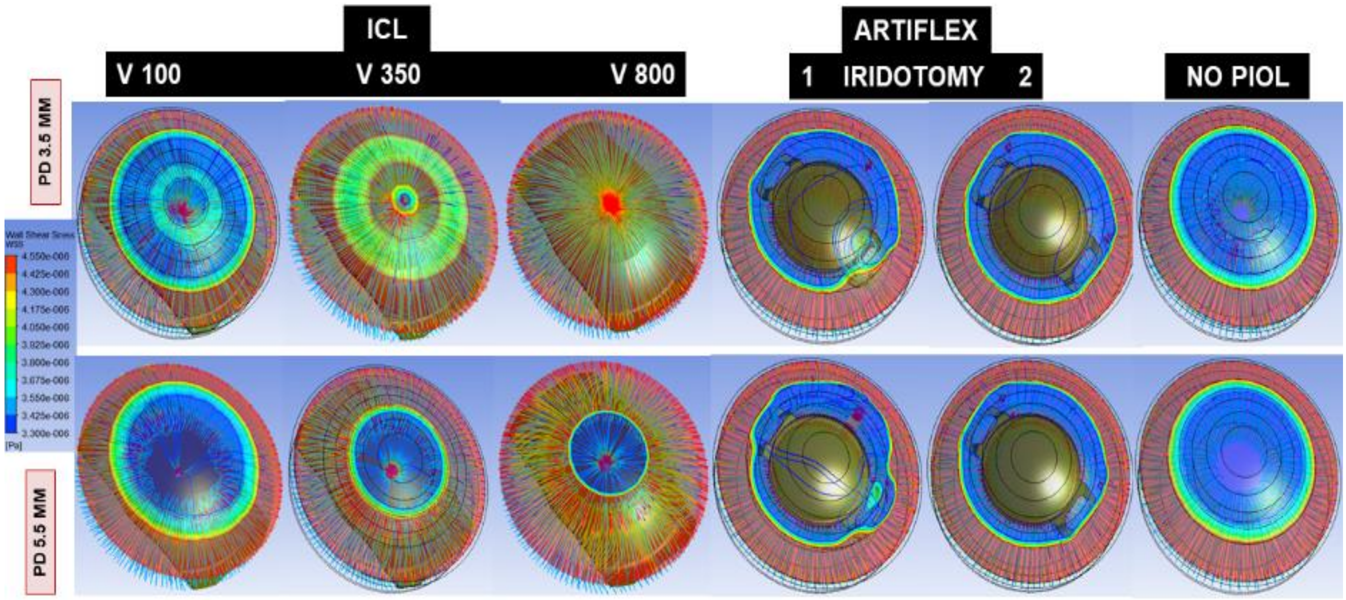

Supporting information, figure 2: Wall shear stress on the corneal endothelium produced by aqueous humour flow through the central hole (ICL) or iridotomy (Artiflex) according to pupil diameter (PD= 3.5 to 5.5 mm), ICL vault (V= 100, 350, 800) and number of Artiflex iridotomies (1 or 2).

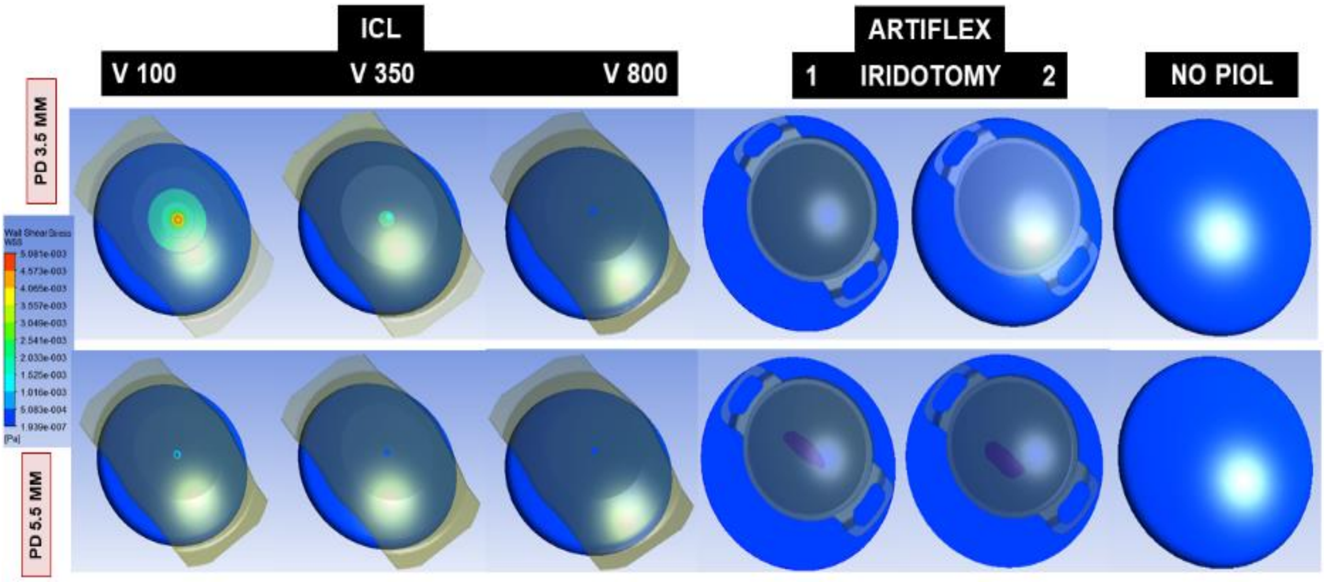

Supporting information, figure 3: Wall shear stress on the crystalline lens produced by aqueous humour flow through the central hole (ICL) or iridotomy (Artiflex) according to pupil diameter (PD= 3.5 to 5.5 mm), ICL vault (V= 100, 350, 800) and number of Artiflex iridotomies (1 or 2).

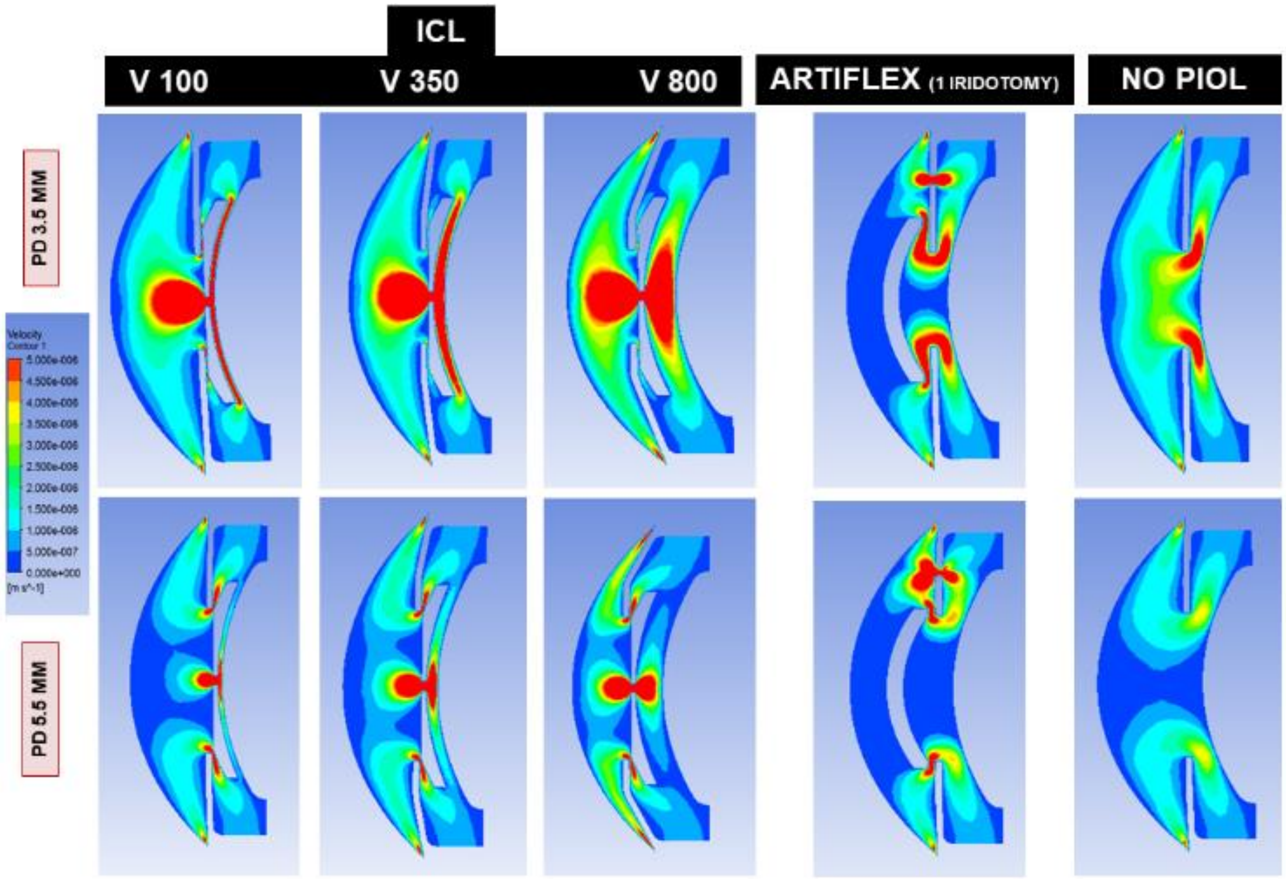

Supporting information, figure 4: Aqueous humour flow velocity through the central hole (ICL) or iridotomy (Artiflex) in the vertical plane according to pupil diameter (PD= 3.5 to 5.5 mm), ICL vault (V= 100, 350, 800) and number of Artiflex iridotomies (1 or 2).
